# Altered functional connectivity of cerebellar dentate nucleus in peak-dose dyskinesia in Parkinson’s disease

**DOI:** 10.3389/fnagi.2022.943179

**Published:** 2022-08-02

**Authors:** Heng Zhang, Lina Wang, Caiting Gan, Xingyue Cao, Min Ji, Huimin Sun, Yongsheng Yuan, Kezhong Zhang

**Affiliations:** Department of Neurology, The First Affiliated Hospital of Nanjing Medical University, Nanjing, China

**Keywords:** Parkinson’s disease, peak-dose dyskinesia, dentate nucleus, resting-state functional magnetic resonance imaging, functional connectivity, voxel-based morphometry analysis

## Abstract

The cerebellum is associated with the emergence of levodopa-induced dyskinesia (LID) in Parkinson’s disease (PD), yet the neural mechanism remains obscure. Our aim was to ascertain the role of functional connectivity (FC) patterns of the cerebellar dentate nucleus (DN) in the pathogenesis of peak-dose dyskinesia in PD. Twenty-three peak-dose dyskinetic PD patients, 27 non-dyskinetic PD patients, and 36 healthy controls (HCs) were enrolled and underwent T1-weighted and resting-state functional magnetic resonance imaging (rs-fMRI) scans after dopaminergic medication intake. We selected left and right DN as the regions of interest and then employed voxel-wise FC analysis and voxel-based morphometry analysis (VBM). The correlations between the altered FC pattern and clinical scores were also examined. Finally, receiver operating characteristic (ROC) curve analysis was performed to assess the potential of DN FC measures as a feature of peak-dose dyskinesia in PD. Dyskinetic PD patients showed excessively increased FC between the left DN and right putamen compared with the non-dyskinetic. When compared with controls, dyskinetic PD patients mainly exhibited increased FC between left DN and bilateral putamen, left paracentral lobule, right postcentral gyrus, and supplementary motor area. Additionally, non-dyskinetic PD patients displayed increased FC between left DN and left precentral gyrus and right paracentral lobule compared with controls. Meanwhile, increased FC between DN (left/right) and ipsilateral cerebellum lobule VIII was observed in both PD subgroups. However, no corresponding alteration in gray matter volume (GMV) was found. Further, a positive correlation between the *z*-FC values of left DN-right putamen and the Unified Dyskinesia Rating Scale (UDysRS) was confirmed in dyskinetic PD patients. Notably, ROC curve analyses revealed that the *z*-FC values of left DN-right putamen could be a potential neuroimaging feature identifying dyskinetic PD patients. Our findings demonstrated that the excessively strengthened connectivity of DN-putamen might contribute to the pathophysiological mechanisms of peak-dose dyskinesia in PD.

## Introduction

Levodopa-induced dyskinesia (LID), which is characterized by abnormal, involuntary and hyperkinetic movement, occurs in nearly 40% of Parkinson’s disease (PD) patients after 4–6 years of levodopa treatment (Ahlskog and Muenter, 2001; Hutny et al., 2021). Peak-dose dyskinesia, the most common genre of LID, appears when the brain dopamine concentration reaches its peak (Espay et al., 2018). However, the precise pathophysiological processes underlying peak-dose dyskinesia remain obscure. Numerous structural and resting-state functional magnetic resonance imaging (rs-fMRI) studies have accentuated the major role of the anomalous brain functional network in LID, which includes the inferior frontal gyrus (IFG), pre-supplementary motor area, and basal ganglia (BG) (Cerasa et al., 2011, 2013, 2015; Herz et al., 2015, 2016; Gan et al., 2020). Overall, the neural mechanisms of peak-dose dyskinesia in PD are implicated in aberrant (Cerasa et al., 2015; Herz et al., 2015, 2016) and uncoordinated (Gan et al., 2020) dopaminergic modulation of cortico-striatal connectivity, beyond the classical BG dysfunctions model.

Aside from the classical motor control loop (cortico-BG-thalamocortical), the cerebellum is another important subcortical structure involved in motor control and sensorimotor information processing (Sathyanesan et al., 2019). Studies in primates (Hoshi et al., 2005; Bostan et al., 2010) and humans (Milardi et al., 2016) have invoked reciprocal links between the BG and cerebellum. Seidel et al. (2017) found that patients suffering from PD had extensive pathology of alpha-synuclein inclusion in the cerebellum, in particular, with mild to moderate aggregation in the deep cerebellar nuclei (such as dentate nuclei). Thus, cerebellar abnormalities in PD might be associated with motor dysfunction. In addition, neuroimaging studies found that altered functional connectivity (FC) between the motor cerebellum and left IFG in patients with *de novo* PD could increase susceptibility to LID (Yoo et al., 2019). Koch et al. (2009) found that continuous theta burst stimulation (cTBS) over the bilateral cerebellum could alleviate dyskinesias markedly and persistently. Kishore and Popa (2014) proposed that abnormal functional alterations in the cerebellum of LID patients impaired their ability to modulate the plasticity of the motor cortex and sensorimotor integration. Altogether, accumulating evidence suggests that the cerebellum has a unique effect on the pathophysiology of LID.

The dentate nucleus (DN), through which most cerebellar signals are projected to the thalamus or motor cortex (Hoshi et al., 2005), is widely used as a region of interest (ROI) to explore the motion control role of the cerebellum (Liu et al., 2013; Ma et al., 2015; Wang N. et al., 2019). Many hypotheses have been proposed regarding the role of altered FC patterns of DN in PD, which may be a mechanism to compensate for the defects of the BG-thalamocortical loop (Liu et al., 2013), or related to the pathogenesis of PD tremor (Ma et al., 2015) or the basis of dysfunction of the somatosensory network in PD (Liu et al., 2013). Combined with the special status in motor function of the DN, we hypothesized that altered FC patterns of DN might participate in the pathophysiological mechanisms of peak-dose dyskinesia in PD. This research used resting-state seed-based FC analysis to explore the DN functional network since FC describes temporal dependencies between neuronal activation patterns in anatomically separated brain regions, providing a method to dig out brain network abnormalities (van den Heuvel and Hulshoff Pol, 2010). Thus, we conducted FC-MRI analyses in PD patients with and without peak-dose dyskinesia and healthy controls (HCs) to investigate this possibility. Furthermore, voxel-based morphometry analysis (VBM) was conducted to ascertain whether or not the brain regions with altered FC were caused by alterations in gray matter volume (GMV).

## Materials and methods

### Subjects

Initially, a total of 60 patients diagnosed with idiopathic PD judging by the Movement Disorder Society (MDS) Clinical Diagnostic Criteria for PD (Postuma et al., 2015) were recruited from the Neurology Unit of the First Affiliated Hospital of Nanjing Medical University, all of whom were right-handed, the unilateral onset of PD and without a family history of PD. Additionally, they had to fulfill the following inclusion criteria: (1) a minimum continuous use of antiparkinsonian drugs including levodopa for half year and no adjustment of medication regimen for 4 weeks at least; (2) presence or absence of peak-dose dyskinesia rather than off-period dyskinesia or diphasic dyskinesia, following an acute levodopa test, observed by two experienced neurologists at the last visit (1 week before the MRI scans); (3) no contraindications to MRI scans; (4) no cognitive impairment [Mini-mental State Examination (MMSE) score > 24] (Crum et al., 1993); (5) no proof of acute or chronic neurological diseases, such as traumatic brain injury, vascular brain lesions; (6) no intake of drugs that could induce cerebral functional change, such as antidepressants, anxiolytics, or antipsychotics; (7) no inordinate movement artifacts (head motions exceeding 2.5 mm of translation or 2.5° of rotation in any direction) during fMRI scans. By these criteria, four PD patients were ruled out from the study due to contraindications to MRI scanning, and another six patients were excluded owing to head movement artifacts. Ultimately, 23 PD patients with peak-dose dyskinesia and 27 patients without dyskinesia were enrolled. Meanwhile, 36 healthy right-handed elderly with matched age, sex, and years of education, without a history of neurological or psychiatric disorders, were recruited as HCs.

PD patients had an obligation to keep diaries 1 week before the start of the experiment to record the beginning and failure of the medicine, as reported in prior trials (Gan et al., 2020; Shen et al., 2020) to establish the timing of MRI scans. Notably, dyskinetic PD patients were additionally required to document the emergence and remission of dyskinetic symptoms to judge the time of LID onset. After taking their regular morning dose of antiparkinsonian medications, all patients received an MRI scan. MRI scans were performed when dyskinetic PD patients were about to develop dyskinetic symptoms, while non-dyskinetic PD patients were performed when they responded to antiparkinsonian medications as expected. During the MRI scan, an expert neurologist remained in the scanning room to observe the subjects and stopped the scan immediately at the onset of abnormal involuntary movements. Fortunately, our participants were not in the circumstance mentioned above. The Unified Dyskinesia Rating Scale (UDysRS) (Goetz et al., 2008) was used to promptly assess the severity of dyskinetic symptoms in PD patients with peak-dose dyskinesia after MRI scans. As it should be, all PD patients underwent clinical assessments, including MMSE, Hoehn and Yahr (H&Y) stage (Hoehn and Yahr, 1967), and Unified Parkinson’s Disease Rating Scale section III (UPDRS-III) (Movement Disorder Society Task Force on Rating Scales for Parkinson’s Disease, 2003). We also calculated each PD individual’s total levodopa equivalent daily dose (LEDD) (Tomlinson et al., 2010).

According to the Helsinki Declaration, the ethics committee of the First Affiliated Hospital of Nanjing Medical University approved the investigation (No. 2016-SRFA-094). Moreover, all participants signed written informed consent before beginning the study.

### Magnetic resonance imaging acquisition

Brain MRI was performed on a 3.0 T Siemens MAGNETOM Verio whole-body MRI scanner (Siemens Medical Solutions, Germany) with an eight-channel, phased-array head coil. Matched foam padding was applied to restrain head movements, and elastic earplugs were applied to avoid interference from the machine noise. All participants were fully informed of inspection processes and precautions ahead of time. They were instructed to stay motionless during the scanning procedure, close their eyes but remain awake, and avoid thinking about anything. To begin, the three-dimensional volumetric magnetization-prepared rapid gradient-echo (3D-MP-RAGE) sequence was employed to obtain 3D T1-weighted anatomical images. Parameter Settings: repetition time (TR): 1 900 ms, echo time (TE): 2.95 ms, flip angle (FA): 9°, field of view (FOV): 230 × 230 mm, slice thickness: 1 mm, slice number: 160, matrix size: 256 × 256, voxel size: 1 × 1 × 1 mm^3^. At last, we obtained the resting-state functional images using an echo-planar imaging sequence. Parameter Settings: TR: 2 000 ms, TE: 21 ms, FA: 90°, FOV: 256 × 256 mm^2^, slice thickness: 3 mm, slice number: 35, no slice gap, total volumes: 240, in-plane matrix: 64 × 64, voxel size: 3 × 3 × 3 mm^3^.

### Image preprocessing

The functional images were preprocessed and analyzed on the MATLAB R2018b platform with DPARSF software.^1^ The initial 10 time points were removed to avoid magnetic saturation effects so that the remaining 230 volumes were reprocessed on the next steps called slice timing and head motion correction (Friston 24). Five dyskinetic PD patients and one non-dyskinetic PD patient with head motions over 2.5 mm of translation or 2.5° of rotation in any direction were eliminated. Mean framewise displacement (FD) values were further calculated, analysis of which revealed no difference among the three groups (*F* = 0.392, *p* = 0.822, Table 1). Skull stripping was applied so that we could perform co-registration of individual T1 structural and functional images precisely. White matter signals, cerebrospinal fluid signals, and six head motion profiles were also regressed out. High-pass temporal filtering (0.01–0.08 Hz) was employed to eliminate low-frequency drifts and high-frequency physiological noise. The filtered images were spatially normalized into the Montreal Neurological Institute (MNI) standard space by the DARTEL technique (resampling voxel size = 3 × 3 × 3 mm^3^) and then smoothed using a Gaussian kernel of 6 mm full-width at half-maximum (FWHM). Linear trend was removed at last.

**TABLE 1:** Demographic and clinical characteristics of all subjects.

Characteristics	Dyskinetic	Non-dyskinetic	Controls	*F/x^2^/z* value	*p*-value
N	23	27	36		
Age (y)	59.26 ± 9.34	58.63 ± 8.01	62.11 ± 5.88	1.89	0.158^a^
Sex (F/M)	8/15	14/13	24/12	5.78	0.056^b^
Education (y)	10.26 ± 3.93	9.74 ± 3.54	11.61 ± 2.92	5.66	0.059^c^
MMSE	28.57 ± 1.44	28.63 ± 1.21	29.14 ± 0.99	3.29	0.193^c^
Mean FD	0.11 ± 0.06	0.11 ± 0.05	0.12 ± 0.05	0.39	0.822^c^
Initial side of onset of motor symptoms (R/L)	9/14	10/17	NA	0.02	0.879^b^
Disease duration (y)	7.48 ± 3.36	6.19 ± 3.03	NA	−1.69	0.091^d^
H-Y stage (ON state)	2.30 ± 0.47	2.07 ± 0.58	NA	−1.72	0.086^d^
UPDRS-III (ON state)	18.39 ± 9.07	17.85 ± 11.87	NA	−0.63	0.526^d^
LEDD, mg/d	669.40 ± 306.24	652.19 ± 176.73	NA	−0.05	0.961^d^
UDysRS	26.35 ± 19.44	NA	NA		

Values are presents as the mean ± standard deviation.

M, male; F, female; y, year; R, right; L, left; MMSE, Mini Mental State Examination; Mean FD, Mean framewise displacement; H-Y stage, Hoehn and yahr clinical rating scale; UPDRS, Unified Parkinson’s disease rating scale; LEDD, levodopa equivalent daily dose; UDysRS, Unified Dyskinesia Rating Scale; NA, not applicable.

^a^One-way ANOVA.

^b^Chi-square test.

^c^Kruskal-Wallis.

^d^Mann-Whitney U.

p < 0.05 was considered statistically significant.

### Defining region of interests and voxel-wise functional connectivity analysis

**FIGURE 1:**
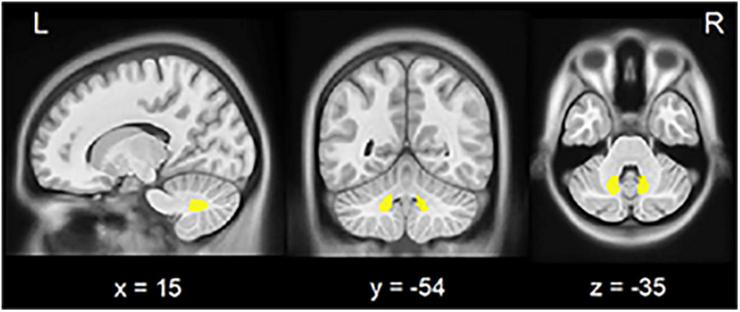
Seed region in the cerebellar dentate nucleus. View of the generated left and right (yellow) dentate nucleus superimposed to the spatially unbiased non-linear brain template (VS Fonov et al., 2010). L, left; R, right.

The left and right DN were separately derived from the Anatomy toolbox (Diedrichsen et al., 2011) and resliced into MNI space (Figure 1). Pearson’s correlation coefficients (*r*) between averaged time courses in each ROI and those in the whole brain were calculated in a voxel-wise manner using DPARSF software. The individual *r*-maps were converted into *z*-maps by Fisher’s *r* to *z* transformation to enhance normality.

One-way factorial analysis of covariance (ANCOVA) was engaged to examine differences in FC patterns of DN among the three groups, using age, sex, education, MMSE scores, and mean FD as covariates. We used Gaussian random field (GRF) correction with voxel-level *p*-value < 0.01 and cluster-level *p*-value < 0.01 as multiple comparison correction (two-tailed). Voxels showing significant differences in the ANCOVA analysis were applied to *post hoc* two-sample *t*-tests to clarify inter-group differences, using the same covariates as in ANCOVA (GRF correction, voxel-level *p*-value < 0.001, cluster-level *p*-value < 0.01, two-tailed). Considering that ANCOVA had the characteristics of exploratory analysis, we chose different voxel level *p*-values for ANCOVA and *post hoc* two-sample *t*-test in order to reduce the false negative rate. All FC analysis was conducted on DPABI software. GRF correction is a multiple comparison correction method using GRF theory at the cluster level, which can be conducted on DPABI software.

### Voxel-based morphometry analysis

VBM analysis was further conducted to examine whether these detected FC patterns of DN were connected with structural changes. VBM8 tool^2^ in Statistical Parametric Mapping (SPM8^3^) software was applied to detect the difference. We segmented the individual 3D-T1 structural images by a unified segmentation model, and the segmented GM images were then normalized by the MNI template (DARTEL technique, resampling voxel size = 1.5 × 1.5 × 1.5 mm3). Afterward, the normalized GM images were modulated by Jacobian determinant derived from normalization and spatially smoothed using an 8 mm FWHM Gaussian kernel.

Voxel-wise GMV differences were examined among the three groups via one-way ANCOVA on DPABI software, using age, sex, education, MMSE scores, and total intracranial volume (TIV) as covariates. The statistical significance was set at a voxel-level *p*-value < 0.001, and multiple comparisons were corrected by GRF with a cluster-level *p*-value < 0.05 (two-tailed).

Then, in order to detected ROI-based morphometry difference, we extracted the mean GMV from brain regions presenting statistical significance in the above FC inter-group comparison (the left and right DN were included) and performed a general linear model to analyze the difference via SPSS, adjusted by age, sex, education, MMSE scores, and TIV. Correction for multiple comparisons (Bonferroni, *p*-value < 0.05) was used to threshold all analyses.

### Statistical analysis

One-way analysis of variance (ANOVA), Kruskal-Wallis, chi-square test, two-sample *t*-test, and Mann-Whitney *U*-test were used appropriately to assess all subjects’ demographic and neuropsychological characteristics relied on SPSS v25.0 software (IBM, Armonk, NY, United States). Subsequently, the *z*-values of FC from brain areas exhibiting significant differences between dyskinetic and non-dyskinetic PD patients were extracted. One rs-fMRI study uncovered that the cognitive network, not only the motor network, was detected in the cerebellar DN functional network (Bernard et al., 2014). Although no difference in MMSE scores and years of education among the three groups was found in our patients, we intended to exclude the interference of the cognitive network involved in DN. Additionally, growing literature revealed that sex, disease duration, and cumulative levodopa exposure were risk factors for LID (Eusebi et al., 2018; Tran et al., 2018). Based on these, partial correlation analysis of FC results against the UDysRS scores was performed in PD patients with peak-dose dyskinesias, using sex, education, LEDD, disease duration, and MMSE scores as covariates (via SPSS). A two-tailed *p*-value < 0.05 was defined as significant.

ROC curves analysis was further performed to assess whether the extracted brain regions could recognize peak-dose dyskinesias in PD. Cut-off values included specificity, sensitivity, and area AUC. Maximizing Youden’s index was used to choose the optimal cut-off.

## Results

### Demographic and clinical data

The demographic and clinical characteristics of the participants are shown in Table 1. The three groups were matched in terms of age, sex, education, MMSE scores, and mean FD. In addition, no difference in disease duration, UPDRS-III scores, H&Y staging scores, LEDD, or initial side of onset of motor symptoms were found between dyskinetic and non-dyskinetic PD patients.

### Functional connectivity analysis

**FIGURE 2:**
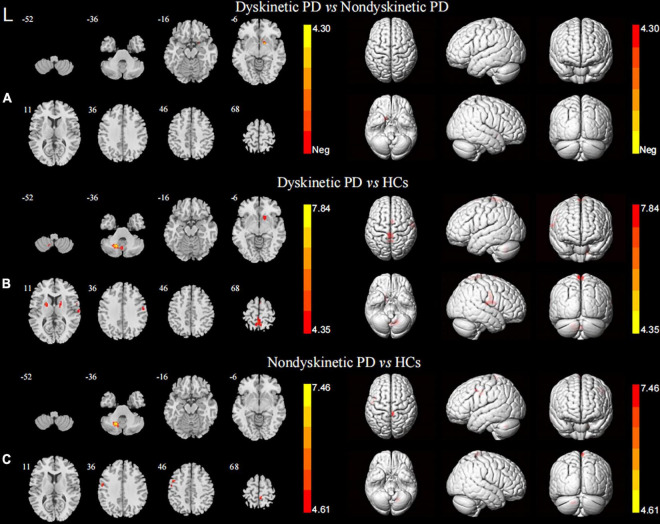
Brain maps for inter-group differences in the left DN functional connectivity. The statistical threshold was set at *p*-value < 0.01, GRF corrected. The brain regions labeled with warm colors represent higher functional connection in the former group. **(A)** Comparison between the dyskinetic and non-dyskinetic PD patients. **(B)** Comparison between the dyskinetic PD patients and HCs. **(C)** Comparison between the non-dyskinetic PD patients and HCs. L, left; PD, Parkinson’s disease; HCs, healthy controls; DN, dentate nucleus; GRF, Gaussian random field (see Table 2 for more detail).

When focusing on the FC patterns of the left DN, we found that the dyskinetic PD patients exhibited significant increased FC between the left DN and the right putamen relative to non-dyskinetic PD patients (Figure 2A and Table 2). Furthermore, in comparison with HCs, significant increased FC were found between left DN and right putamen, left putamen, right postcentral gyrus, left paracentral lobule, right supplementary motor area, left cerebellum lobule VIII in dyskinetic PD patients (Figure 2B and Table 2). And non-dyskinetic PD patients presented significant increased FC between the left DN and right paracentral lobule (MNI_maxima_ *x* = 6, *y* = −36, *z* = 72, *t*-value = 4.61), left precentral gyrus, and left cerebellum lobule VIII compared to controls (Figure 2C and Table 2).

**TABLE 2:** Regions showing significant differences in functional connectivity between the groups.

Brain regions (AAL)	Peak MNI coordinates			Number of voxels	Maximum *t*-value
	
	*x*	*y*	*z*		
ROI: Left dentate nucleus					
Dyskinetic vs. Non-dyskinetic					
Right putamen	15	6	−9	24	4.30
Dyskinetic vs. Controls					
Right putamen	15	12	−3	95	4.35
Left putamen	−21	0	12	64	4.89
Right postcentral gyrus	66	−15	15	84	4.93
Left paracentral lobule	−3	−33	78	129	5.87
Right supply motor area	3	3	78	24	4.97
Left cerebellum lobule VIII	−15	−60	−36	123	7.84
Non-dyskinetic vs. Controls					
Right paracentral lobule	6	−36	72	35	4.61
Left precentral gyrus	−54	−3	36	24	4.46
Left cerebellum lobule VIII	−15	−60	−36	42	7.46
ROI: Right dentate nucleus					
Dyskinetic vs. Non-dyskinetic					
None					
Dyskinetic vs. Controls					
Right cerebellum lobule VIII	12	−60	−39	141	10.46
Non-dyskinetic vs. Controls					
Right cerebellum lobule VIII	12	−60	−36	51	9.44

AAL, Anatomical Automatic Labeling; MNI, Montreal Neurological Institute; The significance of post-hoc t-tests was set at voxel-level p < 0.001, cluster-level p < 0.01, corrected by Gaussian random field; Positive t-value indicates the regions in which the former group had higher functional connectivity than the latter group.

**FIGURE 3:**
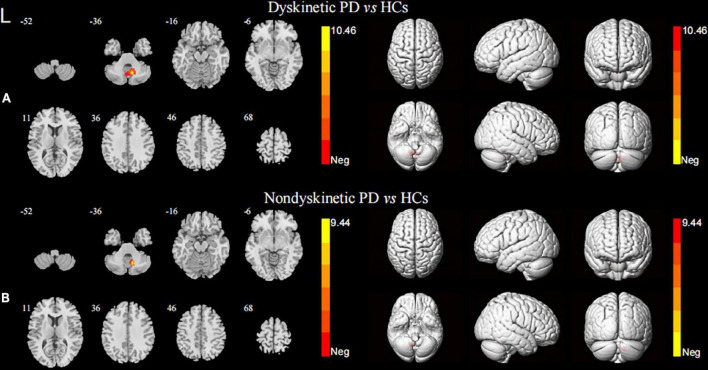
Brain maps for inter-group differences in the right DN functional connectivity. The statistical threshold was set at *p*-value < 0.01, GRF corrected. The brain regions labeled with warm colors represent higher functional connection in the former group. Comparison between the dyskinetic and non-dyskinetic PD patients failed to survive GRF correction. **(A)** Comparison between the dyskinetic PD patients and HCs. **(B)** Comparison between the non-dyskinetic PD patients and HCs. L, left; PD, Parkinson’s disease; HCs, healthy controls; DN, dentate nucleus; GRF, Gaussian random field (see Table 2 for more detail).

In terms of the pattern of the right DN FC, we observed significant enhanced FC between the right DN and the ipsilateral cerebellum lobule VIII in both dyskinetic and non-dyskinetic PD patients relative to HCs (Figure 3A and Table 2). Unfortunately, the comparison between dyskinetic and non-dyskinetic PD patients revealed no statistically significant differences (Figure 3B and Table 2). No decreased FC (left or right DN) in the dyskinetic PD was found compared with non-dyskinetic PD or HCs.

### Voxel-based morphometry analysis

No significant differences in GMV were found among the three groups, indicating that the altered FC patterns of DN were not induced by anatomic changes (Supplementary Table 1).

### Correlation analysis and region of interest curves analysis

**FIGURE 4:**
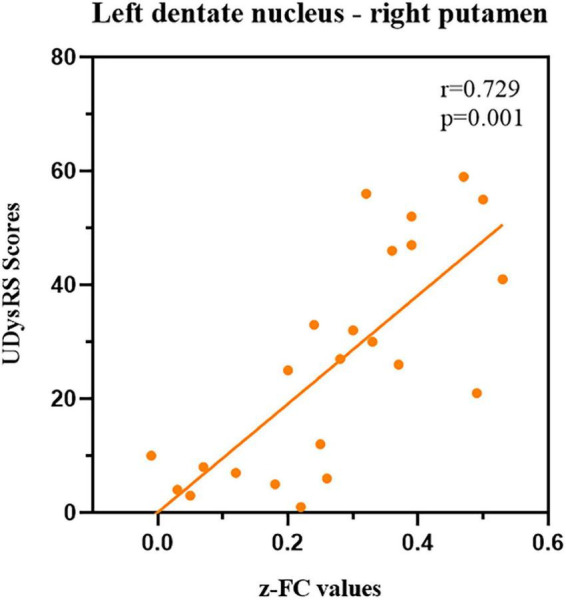
Correlations between *z*-FC values of left DN-right putamen and UDysRS scores during the ON medication state within dyskinetic PD patients. Scatterplots demonstrated that there was a significant positive correlation between the *z*-FC values of left DN-right putamen and UDysRS scores in dyskinetic PD patients. FC, functional connectivity; DN, dentate nucleus; UDysRS, Unified Dyskinesia Rating Scale; PD Parkinson’s disease.

Our correlation analysis indicated that the *z*-FC values of left DN-right putamen (the peak MNI coordinates: 15, 6, and −9; voxel size: 27) were positively associated with the UDysRS scores (*r* = 0.729, *p*-value = 0.001, Figure 4) in dyskinetic PD patients after adjusting sex, education, LEDD, disease duration, and MMSE scores, while no significant association was found between the *z*-values of FC and UPDRS-III or H&Y scale.

**FIGURE 5:**
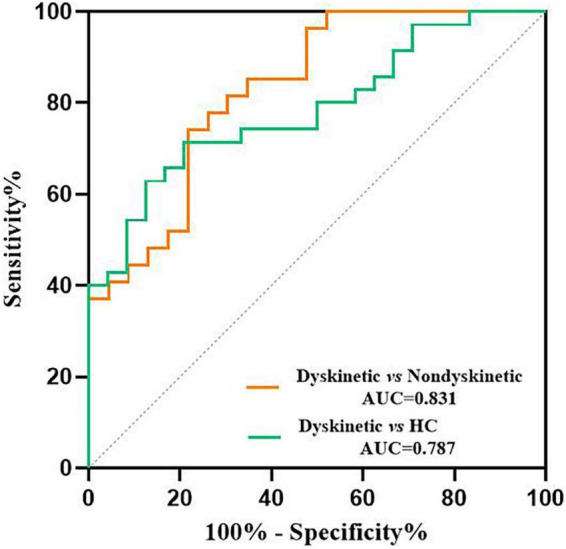
ROC analyses for differentiating different groups. The graph showed that the *z*-FC values of left DN-right putamen shows significant potential as an indicator for distinguishing dyskinetic and non-dyskinetic PD patients (AUC = 0.831, *p* < 0.001) and a primary screening index for separating dyskinetic PD patients from HCs (AUC = 0.787, *p* < 0.001). ROC, receiver operating characteristic; FC, functional connectivity; DN, dentate nucleus; PD, Parkinson’s disease; AUC, area under the curve; HCs, healthy controls (see Table 3 for more detail).

Additionally, receiver operating characteristic (ROC) curves analyses showed that the area under the curve (AUC) of *z*-FC values of left DN-right putamen was 0.831 when separating dyskinetic PD patients from the non-dyskinetic PD patients [95% confidence interval (CI): 0.719–0.943, *p*-value < 0.001] (Table 3 and Figure 5). Meanwhile, the AUC of *z*-FC values of left DN-right putamen was 0.787 when identifying dyskinetic PD patients from HCs (95% CI: 0.674–0.901, *p*-value < 0.001) (Table 3 and Figure 5).

**TABLE 3:** ROC analyses for differentiating different groups.

Brain regions	AUC	*p*-value	95% CI	Sensitivity	Specificity	Cut-off point
**Right putamen**						
Separating dyskinetic from non-dyskinetic	0.831	<0.001*	0.719–0.943	0.783	0.741	0.1706
Separating dyskinetic from controls	0.787	<0.001*	0.674–0.901	0.913	0.583	0.0496
Separating non-dyskinetic from controls	0.492	0.912	0.347–0.637	0.556	0.611	0.0640

ROC, receiver operating characteristic; AUC, area under the curve; CI, confidence interval.

*p < 0.001.

## Discussion

Our fMRI data provided a novel perspective on the neural network model of peak-dose dyskinesia by detecting FC patterns of DN in dyskinetic, non-dyskinetic PD patients and HCs. First, we found that the connectivity of left DN and right putamen was significantly higher in dyskinetic PD patients than in those without dyskinetic symptoms. Further, the *z*-FC values of left DN-right putamen were positively associated with the UDysRS scores, and ROC curves analyses showed that the *z*-FC values of left DN-right putamen could be utilized to separate dyskinetic PD patients from non-dyskinetic PD patients. Second, compared with HCs, PD patients exhibited widespread increased FC between the left DN and the bilateral sensorimotor cortex and increased FC between DN (left/right) and the ipsilateral cerebellum lobule VIII. However, no structural alterations of GMV were observed among the three groups, which suggested that the altered FC patterns of DN were not caused by structural changes.

Although the pathogenesis of LID remains obscure, it is generally believed that LID is attributable to nigrostriatal degeneration and striatal changes connected with chronic dopamine replacement therapy, further inducing abnormal synaptic plasticity of striatal medium spiny neurons (Lerche, 2015). All these combined events altered the neuronal activity in striato-pallidal loops, resulting in increased inhibition of the subthalamic nucleus and excessive stimulation of the motor cortex and ultimately resulting in LID (Donzuso et al., 2020). In addition to the cortico-BG-thalamocortical pathway, the cerebellum might also influence LID via the cerebello-thalamic loop (Kishore and Popa, 2014).

Anatomical studies identified that the cerebellar cortex received projections from the subthalamic nucleus (Bostan et al., 2010), whereas the DN send connections to the striatum via the thalamus (Hoshi et al., 2005). Further, the short-latency of the dentato-thalamo-striatal pathway enabled the BG to integrate time-sensitive cerebellar information and guide the signal of cortico-striatal plasticity to facilitate optimal motor control (Chen et al., 2014). Thus, the cerebellum and BG are anatomically and functionally interconnected, and synaptic modifications or abnormal activity at one node could affect another (Bostan and Strick, 2018). Such observations were in good agreement with our results that dyskinetic PD patients exhibited enhanced connectivity between left DN and right putamen during ON medication state with the presence of clinical dyskinesias, which heralded that peak-dose dyskinesia might result from a pathological interaction in the DN-putamen loop. As we know, the putamen was the most affected region by dopaminergic denervation with the progress of PD (Kish et al., 1988), which was concurrently considered the central target in the pathogenesis of LID (Yang et al., 2021). Herz et al. (2014) observed that dyskinetic PD patients displayed significant overactivation of bilateral putamen after levodopa intake. In particular, an aberrant reinforcement signal in response to levodopa was produced by increased striato-cortical connectivity, contributing to an abnormal motor drive that eventually triggers involuntary movements (Herz et al., 2015). Our previous study concerning topological properties of the white matter structural networks demonstrated that excessively increased node efficiency of right putamen in dyskinetic PD patients transmitted generous anomalous motor drive signals to other regions (Wang L. et al., 2019). Another intriguing evidence detected in a mouse model of cerebellar-induced dystonia demonstrated that the dorsolateral striatum (putamen and caudate) exhibited abnormal high-frequency burst firing, similar to those noted in dystonic patients (Chen et al., 2014). Combined with these findings, we speculated that alterations in neuronal activation patterns and information processing function in left DN, secondary to abnormal putamen signals, might be an essential factor aggravating overactivation in the right putamen and ultimately leading to the emergence of abnormal involuntary movements. In accordance with our hypothesis, inhibition of cerebellar stimulation was verified to alleviate dyskinesias (Koch et al., 2009), which might modulate metabolism in DN and reduce the enhanced DN-putamen connectivity. Besides, our correlation analysis indicated that the severity of dyskinetic symptoms was positively correlated with the *z*-FC values of left DN-right putamen. Furthermore, the ROC curves analyses revealed that the *z*-FC values of left DN-right putamen could distinguish the dyskinetic PD patients from non-dyskinetic PD patients, which could be used as a potential neuroimaging feature identifying peak-dose dyskinesia in PD. This result deserves to be verified in polycentric cohort studies. Overall, our findings of excessively strengthened DN-putamen communication could illustrate that the cerebellum plays an essential role in the neuropathological mechanisms underlying peak-dose dyskinesia, emphasizing that peak-dose dyskinesia might be correlated to the functional alterations of the DN-putamen neural network rather than individual brain nodes.

Compared with controls, both PD subgroup patients displayed remarkably increased FC within the cerebellum (DN-ipsilateral cerebellum lobule VIII) and increased FC between left DN and bilateral sensorimotor cortex after antiparkinsonian drug intake. Neuroimaging studies discovered that cerebellum lobule VIII was predominantly involved in sensorimotor processes and connected with cerebral motor and sensory cortices (Kipping et al., 2013). These results appeared to indicate that PD patients had abnormal sensorimotor functional circuits involving the cerebellum, as described previously (Palmer et al., 2020; Wang S. et al., 2021). Nevertheless, the role of the sensorimotor network involving cerebellum in PD is still controversial. Wang S. et al. (2021) considered that the increased FC in the internal cerebellar networks and sensorimotor networks in PD might compensate for the motor control dysfunction to maintain the integrity of the sensorimotor network. Palmer et al. (2020) proposed that persistent error detection and correction occurring in the cerebellum of PD patients induced the enhanced cerebello-cortical FC. Simioni et al. (2016) advanced a new perspective that the enhanced cerebellar FC in PD patients in mild-to-moderate stages might have a compensatory role but eventually be overwhelmed by the progressive neurodegeneration. Hence, such a compensatory mechanism might have been derailed in our cohort of PD patients in moderate-to-advanced stages. Given that dopaminergic replacement therapy could modulate cerebellar FC patterns (Mueller et al., 2019), our investigation was conducted when PD patients had an expected response to levodopa, in other words, when their motor symptoms ameliorated, which suggested that the increased cerebellar sensorimotor network might be a re-established compensation under the action of drugs. However, no correlation was found between the altered FC patterns of DN (left/right) in PD patients and UPDRS-III scores, which might arise from differences in participant characteristics, such as duration of illness and medication state. Our lack of correlational observations should be interpreted cautiously, and further investigations must be conducted during ON/OFF medication states to elucidate the specific role of sensorimotor circuits involving cerebellum in PD patients.

As we know, lateralization was not rare in PD. Intriguingly, we observed that the alterations of FC patterns were more pronounced between left DN and cerebrum, while no change was found between right DN and cerebrum in both PD subgroups (relative to HCs). Considering that the initial side of onset of motor symptoms in most PD patients in our study appeared on the left side (14 out of 23 in the dyskinetic group and 17 out of 27 in the non-dyskinetic group), it was reasonable that the FC patterns of left DN were altered more. Notably, Martinu et al. (2014) found that the effect of dopaminergic neurodegeneration in the cortico-striatal system was asymmetrical and proposed that levodopa might help improve motor symptoms for the more affected side. In addition, a tendency to appear on the more affected sides (initial unilateral onset) was the hallmark of LID (Espay et al., 2018). Structural MRI revealed that the human cerebellar hemisphere was asymmetric (Schutter, 2019), and a diffusion tensor imaging study uncovered the laterality of cerebellar afferent and efferent pathways in healthy right−handed people (Kim et al., 2019), which provided the neuroanatomical basis of laterality in the cerebellum. A cohort study showed that PD patients with a higher striatal asymmetric index were more susceptible to dyskinesia (Eusebi et al., 2018). Our previous study documented that interhemispheric functional incoordination was correlated to the severity and lateralization of dyskinesias in PD (Gan et al., 2020). Thus, we suspected that the altered FC patterns of left DN we witnessed might underlie the neural mechanisms of hemispheric asymmetries in PD patients with peak-dose dyskinesia, which is worthy of further investigation. Besides, dyskinetic PD patients showed crossed FC between left DN and right putamen in our study compared with non-dyskinetic PD patients. Neuroanatomical studies revealed that the main interconnection between the BG and DN was contralaterally organized (Pelzer et al., 2013; Tacyildiz et al., 2021), making the abnormal left DN-putamen connectivity we witnessed reasonable. However, to our knowledge, this is the first study to explore the altered FC of cerebellar DN in peak-dose dyskinesia in PD, making it difficult to verify whether the peak-dose dyskinesia-related FC between cerebellar DN and putamen was necessarily crossed. In addition, as a previous study described, the FC between putamen and cerebellum were variable in PD patients (sometimes crossed while sometimes ipsilateral connect) (Hou et al., 2016). Considering increased FC between left DN and bilateral putamen were detected compared dyskinetic with non-dyskinetic PD patients in our study, it seems complicated to conclude that dyskinesia-related FC between DN and putamen is crossed, which deserves further exploration.

Apart from a relatively small sample size, several other limitations are worth considering when interpreting these findings. First, as aforementioned, dopaminergic therapies may modulate FC patterns of DN (Mueller et al., 2019), yet our PD patients underwent MRI examination after antiparkinsonian drug intake. Our investigation suggested that LEDD was slightly higher in dyskinetic PD patients than in non-dyskinetic PD patients, which was not statistically significant. Thus, LEDD was also included as covariates during correlation analysis to reduce the nuisance caused by pharmacological treatment. Further, the specific mechanism of peak-dose dyskinesia is more likely to be detected during the ON phase since those dyskinetic symptoms were tightly correlated to dopaminergic therapies. Second, FC is methodologically limited in that it is unable to judge the causal relationship between neuronal activities in two brain regions. Third, we failed to obtain a comprehensive neuropsychological evaluation, limiting our ability to eliminate the interference of confounding factors since the cerebellum also has non-motor functions. Fourth, the amount of antiparkinsonian drugs taken by each PD patient before the MRI scanning was different.

## Conclusion

In conclusion, our study demonstrated that abnormally increased connectivity between the left DN and right putamen was positively associated with the severity of abnormal involuntary movements in dyskinetic PD patients, emphasizing the functional contribution of an excessively strengthened cerebellar-striatal circuit in pathophysiological mechanisms of peak-dose dyskinesia in PD.

## Data availability statement

The original contributions presented in the study are included in the article/Supplementary material, further inquiries can be directed to the corresponding author/s.

## Ethics statement

The studies involving human participants were reviewed and approved by the Ethics Committee of the First Affiliated Hospital of Nanjing Medical University. The patients/participants provided their written informed consent to participate in this study.

## Author contributions

HZ: conception and design of study, acquisition of clinical data, data analysis, writing of the first draft, and revision the manuscript for content. LW: conception and design of study, acquisition of MRI data, data analysis, and revision the manuscript for content. CG: data analysis and acquisition of clinical data. XC, MJ, and HS: acquisition of clinical data and revision the manuscript. YY and KZ: conception and design of study, revision the manuscript for content, study supervision, and obtaining funding. All authors contributed to the article and approved the submitted version.

## Footnotes

1
http://www.restfmri.net/forum/dparsf

2
http://dbm.neuro.uni-jena.de/vbm8/VBM8-Manual.pdf

3
http://www.fil.ion.ucl.ac.uk

## References

[B1] Ahlskog J. E. Muenter M. D. (2001). Frequency of levodopa-related dyskinesias and motor fluctuations as estimated from the cumulative literature. *Mov. Disord.* 16 448–458. 10.1002/mds.1090 11391738

[B2] Bernard J. A. Peltier S. J. Benson B. L. Wiggins J. L. Jaeggi S. M. Buschkuehl M. (2014). Dissociable functional networks of the human dentate nucleus. *Cereb. Cortex* 24 2151–2159. 10.1093/cercor/bht065 23513045 PMC4089384

[B3] Bostan A. C. Dum R. P. Strick P. L. (2010). The basal ganglia communicate with the cerebellum. *Proc. Natl. Acad. Sci. U.S.A.* 107 8452–8456. 10.1073/pnas.1000496107 20404184 PMC2889518

[B4] Bostan A. C. Strick P. L. (2018). The basal ganglia and the cerebellum: Nodes in an integrated network. *Nat. Rev. Neurosci.* 19 338–350. 10.1038/s41583-018-0002-7 29643480 PMC6503669

[B5] Cerasa A. Koch G. Donzuso G. Mangone G. Morelli M. Brusa L. (2015). A network centred on the inferior frontal cortex is critically involved in levodopa-induced dyskinesias. *Brain* 138(Pt 2), 414–427. 10.1093/brain/awu329 25414038

[B6] Cerasa A. Messina D. Pugliese P. Morelli M. Lanza P. Salsone M. (2011). Increased prefrontal volume in PD with levodopa-induced dyskinesias: A voxel-based morphometry study. *Mov. Disord.* 26 807–812. 10.1002/mds.23660 21384430

[B7] Cerasa A. Morelli M. Augimeri A. Salsone M. Novellino F. Gioia M. C. (2013). Prefrontal thickening in PD with levodopa-induced dyskinesias: New evidence from cortical thickness measurement. *Parkinsonism Relat Disord* 19 123–125. 10.1016/j.parkreldis.2012.06.003 22742954

[B8] Chen C. H. Fremont R. Arteaga-Bracho E. E. Khodakhah K. (2014). Short latency cerebellar modulation of the basal ganglia. *Nat. Neurosci.* 17 1767–1775. 10.1038/nn.3868 25402853 PMC4241171

[B9] Crum R. M. Anthony J. C. Bassett S. S. Folstein M. F. (1993). Population-based norms for the Mini-Mental State Examination by age and educational level. *JAMA* 269 2386–2391. 8479064

[B10] Diedrichsen J. Maderwald S. Kuper M. Thurling M. Rabe K. Gizewski E. R. (2011). Imaging the deep cerebellar nuclei: A probabilistic atlas and normalization procedure. *Neuroimage* 54 1786–1794. 10.1016/j.neuroimage.2010.10.035 20965257

[B11] Donzuso G. Agosta F. Canu E. Filippi M. (2020). MRI of motor and nonmotor therapy-induced complications in Parkinson’s disease. *Mov. Disord.* 35 724–740. 10.1002/mds.28025 32181946

[B12] Espay A. J. Morgante F. Merola A. Fasano A. Marsili L. Fox S. H. (2018). Levodopa-induced dyskinesia in Parkinson disease: Current and evolving concepts. *Ann. Neurol.* 84 797–811. 10.1002/ana.25364 30357892

[B13] Eusebi P. Romoli M. Paoletti F. P. Tambasco N. Calabresi P. Parnetti L. (2018). Risk factors of levodopa-induced dyskinesia in Parkinson’s disease: Results from the PPMI cohort. *NPJ Parkinsons Dis.* 4:33. 10.1038/s41531-018-0069-x 30480086 PMC6240081

[B14] Gan C. Wang M. Si Q. Yuan Y. Zhi Y. Wang L. (2020). Altered interhemispheric synchrony in Parkinson’s disease patients with levodopa-induced dyskinesias. *NPJ Parkinsons Dis.* 6:14. 10.1038/s41531-020-0116-2 32665973 PMC7343784

[B15] Goetz C. G. Nutt J. G. Stebbins G. T. (2008). The unified dyskinesia rating scale: Presentation and clinimetric profile. *Mov. Disord.* 23 2398–2403. 10.1002/mds.22341 19025759

[B16] Herz D. M. Haagensen B. N. Christensen M. S. Madsen K. H. Rowe J. B. Lokkegaard A. (2014). The acute brain response to levodopa heralds dyskinesias in Parkinson disease. *Ann. Neurol.* 75 829–836. 10.1002/ana.24138 24889498 PMC4112717

[B17] Herz D. M. Haagensen B. N. Christensen M. S. Madsen K. H. Rowe J. B. Lokkegaard A. (2015). Abnormal dopaminergic modulation of striato-cortical networks underlies levodopa-induced dyskinesias in humans. *Brain* 138(Pt 6), 1658–1666. 10.1093/brain/awv096 25882651 PMC4614130

[B18] Herz D. M. Haagensen B. N. Nielsen S. H. Madsen K. H. Lokkegaard A. Siebner H. R. (2016). Resting-state connectivity predicts levodopa-induced dyskinesias in Parkinson’s disease. *Mov. Disord.* 31 521–529. 10.1002/mds.26540 26954295 PMC5069605

[B19] Hoehn M. M. Yahr M. D. (1967). Parkinsonism: Onset, progression and mortality. *Neurology* 17 427–442. 10.1212/wnl.17.5.427 6067254

[B20] Hoshi E. Tremblay L. Feger J. Carras P. L. Strick P. L. (2005). The cerebellum communicates with the basal ganglia. *Nat. Neurosci.* 8 1491–1493. 10.1038/nn1544 16205719

[B21] Hou Y. B. Yang J. Luo C. Y. Ou R. W. Song W. Liu W. L. (2016). Patterns of striatal functional connectivity differ in early and late onset Parkinson’s disease. *J. Neurol.* 263 1993–2003. 10.1007/s00415-016-8211-3 27394147

[B22] Hutny M. Hofman J. Klimkowicz-Mrowiec A. Gorzkowska A. (2021). Current knowledge on the background, pathophysiology and treatment of levodopa-induced dyskinesia-literature review. *J. Clin. Med.* 10:4377. 10.3390/jcm10194377 34640395 PMC8509231

[B23] Kim Y. Im S. Kim S. H. Park G. Y. (2019). Laterality of cerebellar afferent and efferent pathways in a healthy right-handed population: A diffusion tensor imaging study. *J. Neurosci. Res.* 97 582–596. 10.1002/jnr.24378 30582195

[B24] Kipping J. A. Grodd W. Kumar V. Taubert M. Villringer A. Margulies D. S. (2013). Overlapping and parallel cerebello-cerebral networks contributing to sensorimotor control: An intrinsic functional connectivity study. *Neuroimage* 83 837–848. 10.1016/j.neuroimage.2013.07.027 23872155

[B25] Kish S. J. Shannak K. Hornykiewicz O. (1988). Uneven pattern of dopamine loss in the striatum of patients with idiopathic Parkinson’s disease. Pathophysiologic and clinical implications. *N. Engl. J. Med.* 318 876–880. 10.1056/NEJM198804073181402 3352672

[B26] Kishore A. Popa T. (2014). Cerebellum in levodopa-induced dyskinesias: The unusual suspect in the motor network. *Front. Neurol.* 5:157. 10.3389/fneur.2014.00157 25183959 PMC4135237

[B27] Koch G. Brusa L. Carrillo F. Lo Gerfo E. Torriero S. Oliveri M. (2009). Cerebellar magnetic stimulation decreases levodopa-induced dyskinesias in Parkinson disease. *Neurology* 73 113–119. 10.1212/WNL.0b013e3181ad5387 19597133

[B28] Lerche H. (2015). Can levodopa-induced dyskinesias go beyond the motor circuit? *Brain* 138(Pt 2), 240–242. 10.1093/brain/awu357 25627235

[B29] Liu H. Edmiston E. K. Fan G. Xu K. Zhao B. Shang X. (2013). Altered resting-state functional connectivity of the dentate nucleus in Parkinson’s disease. *Psychiatry Res.* 211 64–71. 10.1016/j.pscychresns.2012.10.007 23352277

[B30] Ma H. Chen H. Fang J. Gao L. Ma L. Wu T. (2015). Resting-state functional connectivity of dentate nucleus is associated with tremor in Parkinson’s disease. *J. Neurol.* 262 2247–2256. 10.1007/s00415-015-7835-z 26159100

[B31] Martinu K. Nagano-Saito A. Fogel S. Monchi O. (2014). Asymmetrical effect of levodopa on the neural activity of motor regions in PD. *PLoS One* 9:e111600. 10.1371/journal.pone.0111600 25369523 PMC4219727

[B32] Milardi D. Arrigo A. Anastasi G. Cacciola A. Marino S. Mormina E. (2016). Extensive direct subcortical cerebellum-basal ganglia connections in human brain as revealed by constrained spherical deconvolution tractography. *Front. Neuroanat.* 10:29. 10.3389/fnana.2016.00029 27047348 PMC4796021

[B33] Movement Disorder Society Task Force on Rating Scales for Parkinson’s Disease (2003). The Unified Parkinson’s Disease Rating Scale (UPDRS): Status and recommendations. *Mov. Disord.* 18 738–750. 10.1002/mds.10473 12815652

[B34] Mueller K. Jech R. Ballarini T. Holiga S. Ruzicka F. Piecha F. A. (2019). Modulatory effects of levodopa on cerebellar connectivity in Parkinson’s disease. *Cerebellum* 18 212–224. 10.1007/s12311-018-0981-y 30298443 PMC6443641

[B35] Palmer W. C. Cholerton B. A. Zabetian C. P. Montine T. J. Grabowski T. J. Rane S. (2020). Resting-state cerebello-cortical dysfunction in Parkinson’s disease. *Front. Neurol.* 11:594213. 10.3389/fneur.2020.594213 33584497 PMC7876057

[B36] Pelzer E. A. Hintzen A. Goldau M. von Cramon D. Y. Timmermann L. Tittgemeyer M. (2013). Cerebellar networks with basal ganglia: Feasibility for tracking cerebello-pallidal and subthalamo-cerebellar projections in the human brain. *Eur. J. Neurosci.* 38 3106–3114. 10.1111/ejn.12314 23879686

[B37] Postuma R. B. Berg D. Stern M. Poewe W. Olanow C. W. Oertel W. (2015). MDS clinical diagnostic criteria for Parkinson’s disease. *Mov. Disord.* 30 1591–1601. 10.1002/mds.26424 26474316

[B38] Sathyanesan A. Zhou J. Scafidi J. Heck D. H. Sillitoe R. V. Gallo V. (2019). Emerging connections between cerebellar development, behaviour and complex brain disorders. *Nat. Rev. Neurosci.* 20 298–313. 10.1038/s41583-019-0152-2 30923348 PMC7236620

[B39] Schutter D. (2019). Hemispheric asymmetries in the human cerebellum. *Cortex* 115 352–356. 10.1016/j.cortex.2018.05.012 29921421

[B40] Seidel K. Bouzrou M. Heidemann N. Kruger R. Schols L. den Dunnen W.F.A. (2017). Involvement of the cerebellum in Parkinson disease and dementia with Lewy bodies. *Ann Neurol* 81, 898–903. 10.1002/ana.24937. 28439961

[B41] Shen Y. T. Yuan Y. S. Wang M. Zhi Y. Wang J. W. Wang L. N. (2020). Dysfunction in superior frontal gyrus associated with diphasic dyskinesia in Parkinson’s disease. *NPJ Parkinsons Dis.* 6:30. 10.1038/s41531-020-00133-y 33145398 PMC7603392

[B42] Simioni A. C. Dagher A. Fellows L. K. (2016). Compensatory striatal-cerebellar connectivity in mild-moderate Parkinson’s disease. *Neuroimage Clin.* 10 54–62. 10.1016/j.nicl.2015.11.005 26702396 PMC4669533

[B43] Tacyildiz A. E. Bilgin B. Gungor A. Ucer M. Karadag A. Tanriover N. (2021). Dentate nucleus: Connectivity-based anatomic parcellation based on superior cerebellar peduncle projections. *World Neurosurg.* 152 E408–E428. 10.1016/j.wneu.2021.05.102 34062299

[B44] Tomlinson C. L. Stowe R. Patel S. Rick C. Gray R. Clarke C. E. (2010). Systematic review of levodopa dose equivalency reporting in Parkinson’s disease. *Mov. Disord.* 25 2649–2653. 10.1002/mds.23429 21069833

[B45] Tran T. N. Vo T. N. N. Frei K. Truong D. D. (2018). Levodopa-induced dyskinesia: Clinical features, incidence, and risk factors. *J. Neural Transm.* 125 1109–1117. 10.1007/s00702-018-1900-6 29971495

[B46] van den Heuvel M. P. Hulshoff Pol H. E. (2010). Exploring the brain network: A review on resting-state fMRI functional connectivity. *Eur. Neuropsychopharmacol.* 20 519–534. 10.1016/j.euroneuro.2010.03.008 20471808

[B47] VS Fonov A.E. RC McKinstry CR Almli DL Collins (2009). Unbiased nonlinear average age-appropriate brain templates from birth to adulthood. *NeuroImage* 47 S102. 10.1016/s1053-8119(09)70884-5

[B48] Wang L. Wang M. Si Q. Yuan Y. Ma K. Gan C. (2019). Altered brain structural topological properties in Parkinson’s disease with levodopa-induced dyskinesias. *Parkinsonism Relat. Disord.* 67 36–41. 10.1016/j.parkreldis.2019.09.022 31621605

[B49] Wang N. Zhang L. Yang H. Liu H. Luo X. Fan G. (2019). Similarities and differences in cerebellar grey matter volume and disrupted functional connectivity in idiopathic Parkinson’s disease and multiple system atrophy. *Neuropsychologia* 124 125–132. 10.1016/j.neuropsychologia.2018.12.019 30590063

[B50] Wang S. Zhang Y. Lei J. Guo S. (2021). Investigation of sensorimotor dysfunction in Parkinson disease by resting-state fMRI. *Neurosci. Lett.* 742:135512. 10.1016/j.neulet.2020.135512 33221477

[B51] Yang K. Zhao X. Wang C. Zeng C. Luo Y. Sun T. (2021). Circuit mechanisms of L-DOPA-Induced Dyskinesia (LID). *Front. Neurosci.* 15:614412. 10.3389/fnins.2021.614412 33776634 PMC7988225

[B52] Yoo H. S. Choi Y. H. Chung S. J. Lee Y. H. Ye B. S. Sohn Y. H. (2019). Cerebellar connectivity in Parkinson’s disease with levodopa-induced dyskinesia. *Ann. Clin. Transl. Neurol.* 6 2251–2260. 10.1002/acn3.50918 31643140 PMC6856615

