# Supplementary material for: Altered functional connectivity of cerebellar dentate nucleus in peak-dose dyskinesia in Parkinson’s disease
Source: Front Aging Neurosci. 2022 Aug 2;14:943179. doi: 10.3389/fnagi.2022.943179 (PMC9400811; doi:10.3389/fnagi.2022.943179)
Supplement: Supplementary file 1 [file Table_1.docx]

# Supplementary Table 1. mean GMV of the brain regions that showing significant differences of FC among three group

| Brain regions | Peak MNI coordinates | | | Voxels | GMV | | | *p* value |
| --- | --- | --- | --- | --- | --- | --- | --- | --- |
|  | ***x*** | ***y*** | ***z*** |  | **Dyskinetic** | **Nondyskinetic** | **Controls** |  |
| Right putamen | 15 | 6 | -9 | 24 | 0.46±0.07 | 0.44±0.07 | 0.46±0.08 | 0.332 |
|  | 15 | 12 | -3 | 95 | 0.28±0.02 | 0.28±0.04 | 0.28±0.03 | 0.697 |
| Left Cerebellum lobule VIII | -15 | -60 | -36 | 123 | 0.33±0.03 | 0.32±0.03 | 0.33±0.04 | 0.245 |
|  |  |  |  | 42 | 0.22±0.02 | 0.22±0.02 | 0.23±0.02 | 0.273 |
| Right Cerebellum lobule VIII | 12 | -60 | -39 | 141 | 0.39±0.04 | 0.38±0.05 | 0.40±0.05 | 0.550 |
|  | 12 | -60 | -36 | 51 | 0.27±0.03 | 0.26±0.03 | 0.27±0.03 | 0.677 |
| Left putamen | -21 | 0 | 12 | 64 | 0.26±0.04 | 0.26±0.04 | 0.27±0.03 | 0.811 |
| Right postcentral gyrus | 66 | -15 | 15 | 84 | 0.42±0.05 | 0.41±0.05 | 0.41±0.05 | 0.489 |
| Right paracentral lobule | 6 | -36 | 72 | 35 | 0.27±0.04 | 0.27±0.07 | 0.28±0.04 | 0.073 |
| Left paracentral lobule | -3 | -33 | 78 | 129 | 0.22±0.02 | 0.22±0.05 | 0.22±0.03 | 0.164 |
| Left precentral gyrus | -54 | -3 | 36 | 24 | 0.49±0.06 | 0.45±0.10 | 0.49±0.05 | 0.06 |
| Right supply motor area | 3 | 3 | 78 | 24 | 0.10±0.02 | 0.10±0.03 | 0.10±0.02 | 0.180 |
| Left Dentate | - | - | - | - | 0.16±0.01 | 0.16±0.01 | 0.16±0.02 | 0.346 |
| Right Dentate | - | - | - | - | 0.20±0.02 | 0.20±0.02 | 0.21±0.03 | 0.679 |

Values are represented as the mean ± standard deviation. The significance of ANCOVA was set at p < 0.05. GMV gray matter volume; FC functional connectivity; MNI Montreal Neurological Institute
